# CircTLK1 modulates sepsis‐induced cardiomyocyte apoptosis via enhancing PARP1/HMGB1 axis–mediated mitochondrial DNA damage by sponging miR‐17‐5p

**DOI:** 10.1111/jcmm.16738

**Published:** 2021-08-19

**Authors:** Yu Qiu, Ying Yu, Xiao‐Mei Qin, Tao Jiang, Yan‐Fang Tan, Wen‐Xian Ouyang, Zheng‐Hui Xiao, Shuang‐Jie Li

**Affiliations:** ^1^ Emergency Center Hunan Children’s Hospital Changsha China; ^2^ Department of Hepatopathy Hunan Children’s Hospital Changsha China

**Keywords:** apoptosis, circTLK1/miR‐17‐5p/PARP1/HMGB1 axis, mitochondrial dysfunction, mtDNA oxidative damage, septic cardiomyopathy

## Abstract

**Introduction:**

Septic cardiomyopathy is a common complication of sepsis with high morbidity and mortality, but lacks specific therapy. This study aimed to reveal the role of circTLK1 and its potential mechanisms in septic cardiomyopathy.

**Materials and Methods:**

The in vitro and in vivo models of septic cardiomyopathy were established. Cell viability and apoptosis were detected by CCK8, TUNEL and flow cytometry, respectively. LDH, CK, SOD, MDA, ATP, 8‐OHdG, NAD+/NADH ratio, ROS level, mitochondrial membrane potential and cytochrome C distribution were evaluated using commercial kits. qRT‐PCR and western blotting were performed to detect RNA and protein levels. Mitochondrial DNA (mtDNA) copy number and transcription were assessed by quantitative PCR. Dual‐luciferase assay, RNA immunoprecipitation and co‐immunoprecipitation were performed to verify the interaction between circTLK1/PARP1 and miR‐17‐5p.

**Results:**

CircTLK1, PARP1 and HMGB1 were up‐regulated in the in vitro and in vivo models of septic cardiomyopathy. CircTLK1 inhibition restrained LPS‐induced up‐regulation of PARP1 and HMGB1. Moreover, circTLK1 knockdown repressed sepsis‐induced mtDNA oxidative damage, mitochondrial dysfunction and consequent cardiomyocyte apoptosis by inhibiting PARP1/HMGB1 axis in vitro and in vivo. In addition, circTLK1 enhanced PARP1 expression via sponging miR‐17‐5p. Inhibition of miR‐17‐5p abolished the protective effects of circTLK1 silencing on oxidative mtDNA damage and cardiomyocyte apoptosis.

**Conclusion:**

CircTLK1 sponged miR‐17‐5p to aggravate mtDNA oxidative damage, mitochondrial dysfunction and cardiomyocyte apoptosis via activating PARP1/HMGB1 axis during sepsis, indicating that circTLK1 may be a putative therapeutic target for septic cardiomyopathy.

## 1 INTRODUCTION

Sepsis, a life‐threatening systemic inflammatory reaction caused by bacterial infection, is recognized as a major cause of death particularly in the older population.^1^ Cardiomyopathy is one of the most life‐threatened complications of sepsis, which occurs in approximately 50% of septic patients and greatly increases the mortality risk of those patients.^2^, ^3^, ^4^ At present, controlling infection and non‐specific supportive care are common treatment strategies for septic cardiomyopathy.^5^ However, there is still a lack of effective therapy for septic cardiomyopathy due to its unclear pathogenesis. Therefore, further exploration of the pathological mechanisms underlying septic cardiomyopathy may contribute to identifying effective therapeutic targets.

Mitochondrial DNA (mtDNA) is a circular DNA molecule that encodes multiple essential proteins participated in oxidative phosphorylation.^6^ Previous studies have suggested that mtDNA is more vulnerable than nuclear DNA to free radicals, such as reactive oxygen species (ROS), owing to its location near the respiratory chain and lack of histone protection.^7^, ^8^ mtDNA damage may decrease the expression of proteins involved in electron transport, which results in excessive ROS production and mitochondrial dysfunction that finally triggers apoptosis.^9^ It has been documented that the intact mtDNA content was significantly decreased in septic animals and patients.^10^, ^11^ In addition, mtDNA damage has been observed in various heart diseases, such as sepsis‐induced cardiac dysfunction,^12^ doxorubicin‐induced cardiomyopathy,^13^ heart failure^14^ and so on. Yao et al showed that the excessive ROS release led to mtDNA damage, which aggravated inflammatory response in sepsis‐induced myocardial injury.^12^ Thus, attenuating mtDNA damage may be a promising approach to treat septic cardiomyopathy.

Circular RNAs (circRNAs) are a novel class of non‐coding RNAs, expressed extensively in multiple tissues and cells. Dysregulation of circRNAs takes part in the pathogenesis of various diseases, including sepsis.^15^ circRNAs were found to be aberrantly expressed in the heart tissues of septic rats with myocardial depression.^16^ However, the biological functions of circRNAs and the related potential mechanisms in septic cardiomyopathy have not been elucidated. Up‐regulation of circTLK1, also known as circ_009932, has been reported to favour the progression of myocardial ischaemia/reperfusion injury in mice.^17^ Given the above background, we speculated that dysregulation of circTLK1 might also participate in septic cardiomyopathy. MicroRNAs (miRNAs) are another kind of non‐coding RNAs. CircRNAs have been reported to function as miRNA ‘sponges’ to repress the expression of miRNAs.^18^ MiR‐17‐5p, as an inflammatory regulator, has been verified to be a player in sepsis‐induced acute lung injury.^19^ More importantly, miR‐17‐5p could inhibit norepinephrine‐induced cardiomyocyte apoptosis via targeting Apaf‐1.^20^ As predicted by starBase database, there is complementarity between miR‐17‐5p and circTLK1 sequences. Therefore, we hypothesized that circTLK1 might contribute to the progression of septic cardiomyopathy via sponging miR‐17‐5p.

Poly(ADP‐ribose) polymerase 1 (PARP1), an important member of the PARP family, is an enzyme responsible for DNA repair. Upon DNA damage, PARP1 is recruited to the damaged sites and catalyses the synthesis of polymer termed poly(ADP‐ribose) chains.^21^ PARP1 also plays crucial roles in controlling inflammatory processes via transcriptional regulation of inflammatory mediators.^22^ Moreover, PARP1 has been demonstrated to be involved in mitochondrial homeostasis and mtDNA maintenance due to its location in the nucleus.^23^ High mobility group box 1 protein (HMGB1) is a protein extensively expressed in eukaryotic cells. The release of HMGB1 leads to injury of multiple organs during sepsis.^24^ A previous study showed that HMGB1 could cause inflammatory response during the progression of hepatocellular carcinoma through activating TLR9 signalling pathway via interaction with cytosol mtDNA.^25^ Recent evidence has suggested that PARP1 facilitated LPS‐induced HMGB1 release via acetylation modification.^26^, ^27^ Besides, the interaction between PARP1 and HMGB1 proteins has also been reported.^28^, ^29^

CircRNAs have been demonstrated to compete with mRNAs for miRNAs binding, which inhibit downstream gene expression.^30^ The biological functions of circRNA/miRNA/mRNA axis in sepsis have been revealed.^31^, ^32^ More importantly, bioinformatics analysis predicted the complementarity between miR‐17‐5p and circTLK1/PARP1. Therefore, we speculated that circTLK1 might sponge miR‐17‐5p to favour the pathological progression of septic cardiomyopathy via regulating PARP1/HMGB1 axis–mediated mtDNA oxidative damage.

In the present study, we investigated the biological functions of circTLK1 in the in vitro and in vivo models of septic cardiomyopathy. Here, we found that circTLK1 was up‐regulated in LPS‐stimulated cardiomyocytes. CircTLK1 sponged miR‐17‐5p to facilitate oxidative stress–mediated mtDNA damage, mitochondrial dysfunction and subsequent apoptosis in sepsis‐induced cardiomyopathy in vitro and in vivo via activating PARP1/HMGB1 axis. Targeting circTLK1/miR‐17‐5p/PARP1/HMGB1 axis may become a new strategy for septic cardiomyopathy treatment.

## 2 MATERIALS AND METHODS

### 2.1 Cell culture and treatment

Human cardiomyocytes (HCMs) were purchased from American Type Culture Collection (Manassas, VA, USA). The cells were maintained in Dulbecco's modified Eagle's medium (Sigma‐Aldrich) supplemented with 10% foetal bovine serum at 37°C under 5% CO_2_. HCM cells were exposed to various concentrations of LPS (0.1, 1, 2, 5, 10 μg/mL, Sigma‐Aldrich) for 24 hours. To inhibit PARP1 expression, HCM cells were pre‐treated with PJ34 (PARP1 inhibitor, 30 µmol/L; AdooQ BioScience) for 1 hour before the stimulation with LPS.

The short hairpin RNA targeting circTLK1 (sh‐circTLK1), negative control shRNA (sh‐NC), miR‐17‐5p mimics, mimics negative control (mimics NC), miR‐17‐5p inhibitor and inhibitor negative control (inhibitor NC) were purchased from GenePharma. HCM cells were seeded into 96‐well plates and cultured to 70% confluence. Then, the cells were transfected with above oligonucleotides for 48 hours using Lipofectamine 2000 (Thermo Fisher).

### 2.2 Cell counting kit‐8 assay

The viability of cardiomyocytes was examined by cell counting kit‐8 (CCK8) assay. Briefly, the HCM cells with various treatments were inoculated in 96‐well plates and added with 10 μL CCK8 solution (Dojindo). After incubation for 1 hour at 37°C in the incubator, the absorbance value was detected at 450 nm using a microplate reader (ALLSHENG).

### 2.3 RNA extraction and quantitative real‐time PCR (qRT‐PCR)

Total RNA was extracted from HCM cells or myocardial tissues using an *EasyPure* ^®^ RNA Kit (TransGen Biotech) following the manufacturer’s instructions. Complementary deoxyribose nucleic acid (cDNA) was synthesized with a *TransScript* ^®^ One‐Step RT‐PCR SuperMix (TransGen Biotech), followed by quantitative PCR using SYBR Green Realtime PCR Master Mix (SinoBio). The relative expression levels of genes normalized to β‐actin or U6 were calculated using $2^{‐\Delta \Delta C_{t}}$ method.

### 2.4 Western blotting

Total protein was extracted from cells or tissues using RIPA buffer (Beyotime) and quantified with an *Easy* II Protein Quantitative Kit (TransGen Biotech). The protein samples were subjected to SDS‐PAGE and then transferred onto polyvinylidene difluoride membranes. The membranes were blocked with 5% skim milk for 1 hour and incubated with primary antibodies against PARP1 (1:1000, PB9309, Boster Biological Technology), HMGB1 (1:1000, A00066‐1, Boster Biological Technology), Bcl‐2 (1:1000, A00040‐2, Boster Biological Technology), Bax (1:500, bs‐0127R, Bioss, Beijing, China), cleaved caspase‐3 (c‐Caspase3; 1:500, BA3257, Boster Biological Technology), acetylated lysine (1:500, MA1‐2021, Thermo Fisher), cytochrome C (1:500, bs‐0013R, Bioss), COX IV (1:2000, bsm‐52750R, Bioss) and GAPDH (1:1000, BA2913, Boster Biological Technology) overnight at 4°C. Then, HRP‐conjugated AffiniPure Goat Anti‐rabbit/mouse IgG secondary antibody (1:5000, Boster Biological Technology) was incubated for 1 hour. The *EasySee* ^®^ Western Blot Kit (TransGen Biotech) was used to detect the protein bands.

### 2.5 Measurement of oxidative stress

To determine the intracellular ROS level, the HCM cells were stained with MitoSOX Red (2.5 µmol/L, Thermo Fisher) for 20 minutes. Then, the cells were washed three times with PBS to remove the residual MitoSOX Red. The ROS signals were captured under a fluorescent microscope (Olympus). The activity of superoxide dismutase (SOD) and level of malondialdehyde (MDA) were evaluated using the commercial kits (Jiancheng Institute of Biotechnology) according to the manufacturer’s instructions.

### 2.6 Detection of myocardial markers

The lactate dehydrogenase (LDH) and creatine kinase (CK) levels in the supernatant of cardiomyocytes were measured using the LDH and CK detection kit (Jiancheng Institute of Biotechnology), respectively, according to the manufacturer's instructions.

### 2.7 Detection of ATP level

The ATP level in HCM cells was detected using an ATP Assay Kit (Beyotime). In brief, HCM cells were lysed in lysis buffer and centrifuged at 12 000 *g* for 5 minutes at 4°C to collect the supernatants. Subsequently, 20 µL of supernatants were incubated with 100 µL of working solution for 5 minutes. The luminance was detected using a microplate reader. The ATP level was calculated based on the standard curve.

### 2.8 Detection of NAD+/NADH ratio

NAD+/NADH ratio in HCM cells was determined using a NAD+/NADH Assay Kit with WST‐8 (Beyotime) according to a previous study.^33^ Briefly, HCM cells were lysed in 200 µL of cooled NAD+/NADH extracting solution. After centrifugation at 12 000 *g* for 10 minutes at 4°C, the supernatants were collected and incubated with working solution for 10 minutes at 37°C and then incubated with 10 µL of colour reagent for 30 minutes at 37°C. The absorbance at 450 nm was measured using a microplate reader. The concentrations of NAD+ and NADH were calculated according to the standard curves. Finally, the ratio of NAD+/NADH was calculated.

### 2.9 Measurement of apoptosis

To evaluate apoptosis, the HCM cells with various treatments were collected and stained with Annexin V for 15 minutes and then stained with propidium iodide (PI) for 30 minutes away from light. Immediately after the staining, HCM cells were detected on a flow cytometry.

The apoptosis of HCM cells and myocardial tissues was also detected using a TUNEL Apoptosis Assay Kit (Solarbio, Beijing, China) following the manufacturer's instructions.

### 2.10 Detection of mitochondrial membrane potential (∆Ψm)

JC‐1 dye (MedChemExpress) was used to detect the loss of ∆Ψm. Briefly, HCM cells received different treatments were stained with 1 μg/mL JC‐1 for 15 minutes and then washed with PBS. The fluorescence was observed under a fluorescence microscope. The fluorescence change from red or red‐orange to green indicates the loss of ∆Ψm.

### 2.11 Immunofluorescence staining

The distribution of cytochrome C in HCM cells was observed by immunofluorescence staining. After fixation with 4% paraformaldehyde, the HCM cells were permeabilized with Triton X‐100 and blocked with normal goat serum. Then, HCM cells were incubated with primary antibody cytochrome C (1:100, bs‐0013R, Bioss) at 4°C overnight. Next, Mouse Anti‐Rabbit IgM/Cy3 (1:100, bs‐0369M‐Cy3, Bioss) was incubated for 1 hour. After nuclear staining with DAPI and mitochondrion labelling with mito‐Tracker Green (Beyotime), the fluorescence was observed under a fluorescent microscope.

### 2.12 Co‐immunoprecipitation

The HCM cells were lysed in RIPA buffer supplemented with protease inhibitor cocktail (Beyotime) and then incubated with Protein A/G Magnetic Agarose Beads (Thermo Fisher) for 2 hours at 4°C. Subsequently, the supernatants were collected after centrifugation at 1000 *g* for 5 minutes, followed by incubation with non‐specific IgG (1 μg, A7016; Beyotime) or HMGB1 antibody (10 μg, H9539; Sigma‐Aldrich) overnight at 4°C. After washing with PBS for three times, the protein A/G beads were detected by Western blotting as described above.

### 2.13 8‐hydroxydesoxyguanosine

The 8‐hydroxydesoxyguanosine (8‐OHdG) level in HCM cells and myocardial tissues was detected using the 8‐OHdG ELISA kit (R&D Systems). Briefly, the mitochondria were isolated from HCM cells or myocardial tissues using a cell mitochondria isolation kit (Beyotime) or a tissue mitochondria isolation kit (Beyotime), respectively. Then, the 8‐OHdG level was detected according to the manufacturer’s instructions.

### 2.14 mtDNA copy number and transcription level

To detect the mtDNA copy number, an *EasyPure* ^®^ Genomic DNA Kit (TransGen Biotech) was used to isolate total DNA from HCM cells or myocardial tissues. Then, SYBR Green Realtime PCR Master Mix (SinoBio) was used for quantitative PCR. The mtDNA copy number was calculated as the relative contents of mtDNA (subunit II of cytochrome c oxidase, COII) to nuclear DNA (β‐actin). To determine mitochondrial DNA transcription level, the mRNA expression levels of mtDNA‐encoded ND1, CytB and ATP6 were determined using qRT‐PCR as described above.

### 2.15 Dual‐luciferase reporter system

The wild‐type (WT) or mutant type (MUT) fragments of circTLK1 or the 3’UTR of PARP1 containing complementarity with miR‐17‐5p were inserted into the pGL3 luciferase reporter vector (Promega), namely circTLK1‐WT, circTLK1‐MUT, PARP1‐WT and PARP1‐MUT plasmids, respectively. Subsequently, HCM cells were co‐transfected with the above‐constructed plasmid together with miRNA‐17‐5p mimics or mimic NC using Lipofectamine 2000. After the transfection for 48 hours, the luciferase activity was detected using a Dual‐Lucy Assay Kit (Solarbio).

### 2.16 RNA‐binding protein immunoprecipitation (RIP)

As described previously,^34^, ^35^ the interaction between circTLK1 and miR‐17‐5p was assessed by RIP assay using the RNA‐Binding Protein Immunoprecipitation Kit (Millipore). Briefly, HCM cells transfected with miR‐17‐5p mimic or mimic NC were lysed and incubated with magnetic beads conjugated with Ago2 antibody (10686‐1‐AP, Proteintech) or IgG (Thermo Fisher) for 4 hours at 4°C. IgG was used as the control. Then, the levels of circTLK1 in precipitated RNA was detected by qRT‐PCR.

### 2.17 Rat model of sepsis

Adult male Sprague Dawley rats (bodyweight, 200‐250 g) were purchased from Slac Jingda Laboratory Animal Co., Ltd. These animals were housed with free access to food and water. After acclimatization for 1 week, the rats were randomly divided into four groups: sham, caecal ligation and puncture (CLP), CLP+sh‐circTLK1 and CLP+sh‐circTLK1+PJ34. CLP was performed to induce sepsis. Briefly, after anaesthesia, a midline incision was made to expose the caecum. A ligation was made below the ileocecal valve, the caecum was transversely punctured using a 21‐gauge needle. Then, the bowel was returned to the abdominal cavity and the wound was carefully stitched. The sham rats were subjected to the same abdominal incision but without ligation or punctures. The rats in CLP+sh‐circTLK1+PJ34 group were intraperitoneally injected with PJ34 (30 mg/kg) immediately after the CLP once every 8 hours. The rats in sh‐circTLK1 group were injected with lentiviruses expressing sh‐circTLK1 (Genechem, Shanghai, China) via the caudal vein. At 24 hours after CLP, all rats were killed and the myocardial tissues were collected. All experiments performed were approved by the Animal Care and Use Committee of Hunan Children’s Hospital.

### 2.18 Statistical analysis

Data are shown as mean ± standard deviation (SD). Student’s *t*‐test or one‐way analysis of variance followed by Tukey's test using GraphPad Prism 8 was used to evaluate the differences among two or more groups. Statistically significance was set as *P* value <0.05.

## 3 RESULTS

### 3.1 LPS exposure leads to up‐regulation of PARP1, HMGB1 and circTLK1 oxidative stress damage and apoptosis in HCM cells

**FIGURE 1:**
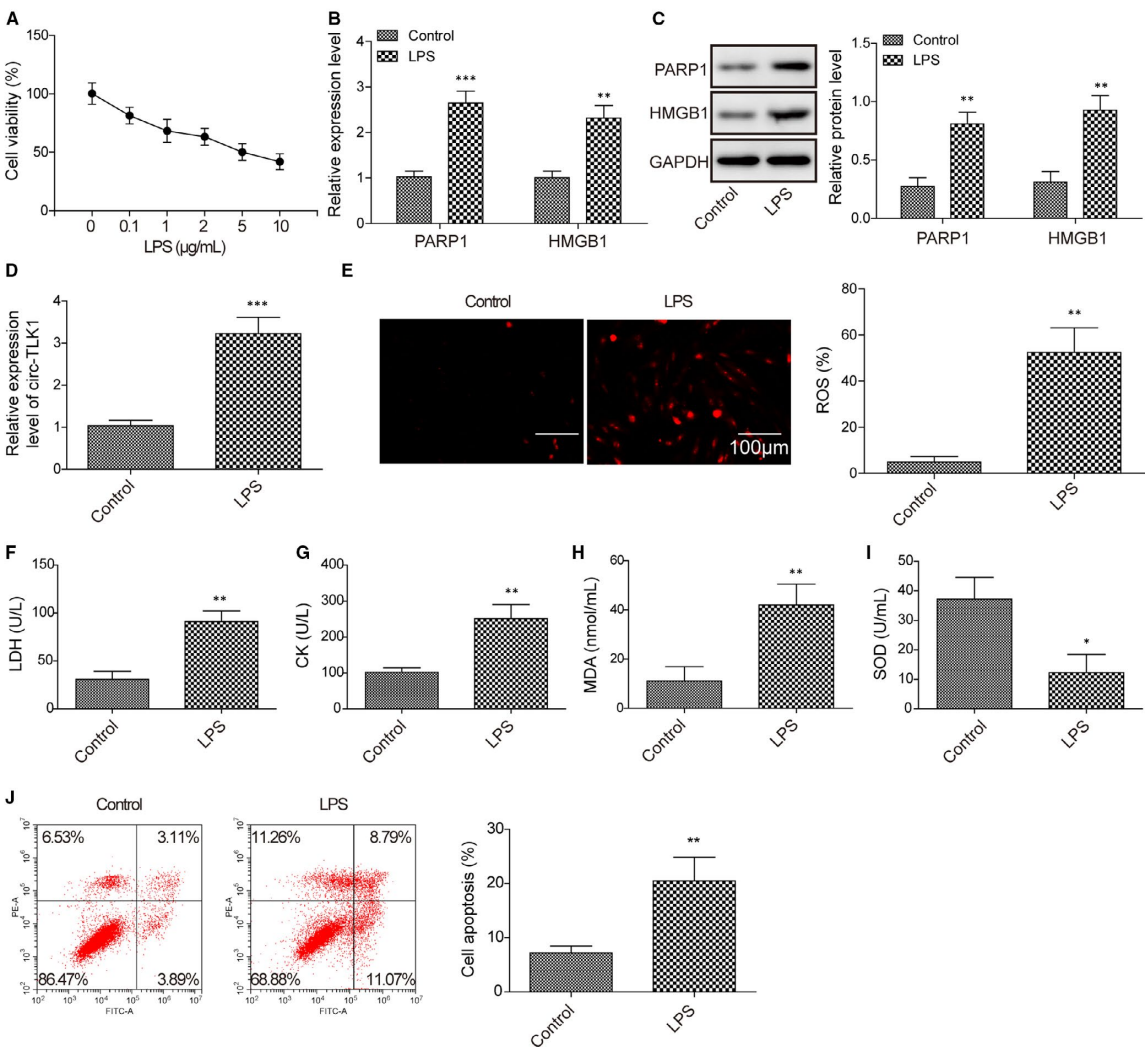
Effect of LPS challenge on expression of PARP1, HMGB1 and circTLK1 oxidative stress and apoptosis in HCM cells. A, The viability of HCM cells after exposure to various concentrations of LPS. B, The mRNA expression of PARP1 and HMGB1 in LPS‐stimulated HCM cells was shown. C, Western blotting for PARP1 and HMGB1 protein levels in HCM cells induced by LPS. D, The expression of circTLK1 in HCM cells. E, The production of ROS in HCM cells. The lactate dehydrogenase (LDH) (F), creatine kinase (CK) (G) levels in the cellular supernatant, and the MDA level (H) and SOD activity (I) in HCM cells were evaluated. J, The apoptosis of HCM cells was assessed. All results from three independent experiments were expressed as mean ± SD. *P < .05; **P < .01; ***P  < .001 vs control group

To evaluate the viability of HCM cells stimulated with various concentrations of LPS, CCK8 was performed. As shown in Figure 1A, LPS exposure significantly reduced the viability of HCM cells in a dose‐dependent manner. Based on this result, 5 μg/mL LPS was selected in subsequent experiments. The mRNA (Figure 1B) and protein (Figure 1C) expression levels of PARP1 and HMGB1 were remarkably increased after LPS stimulation. Furthermore, LPS administration led to up‐regulation of circTLK1 in HCM cells (Figure 1D). In addition, LPS stimulation increased the levels of ROS (Figure 1E), LDH (Figure 1F), CK (Figure 1G), and MDA (Figure 1H), but decreased the activity of SOD (Figure 1I) in HCM cells. Moreover, the apoptotic rate in HCM cells was evidently elevated after challenge with LPS (Figure 1J). The above data indicated that the abnormal up‐regulation of PARP1, HMGB1 and circTLK1 might play a crucial role in LPS‐induced cardiomyocyte injury.

### 3.2 CircTLK1 or PARP1 inhibition restrains LPS‐induced apoptosis in HCM cells

**FIGURE 2:**
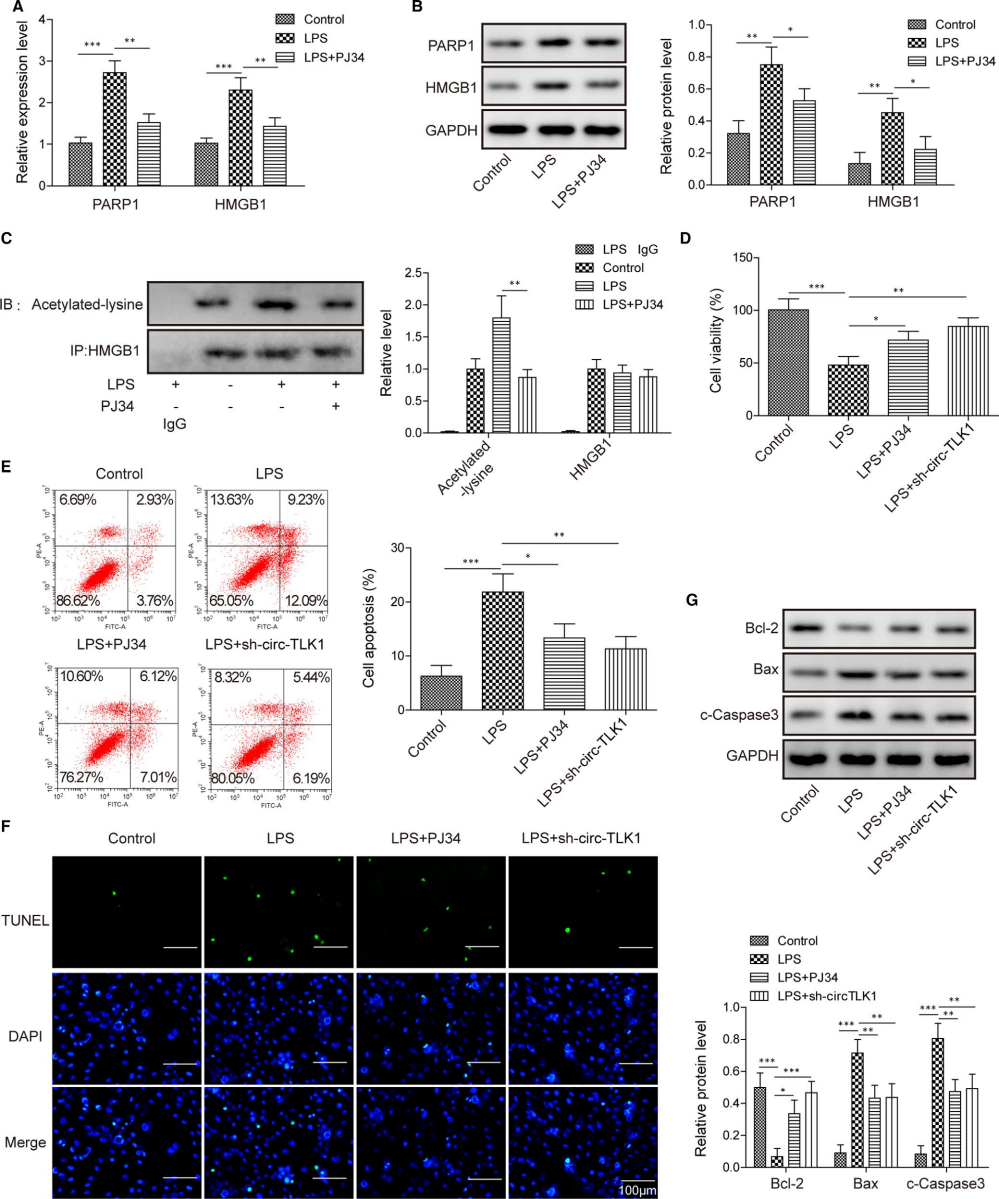
CircTLK1 or PARP1 inhibition restrains LPS‐induced apoptosis in HCM cells. The mRNA (A) and protein (B) levels of PARP1 and HMGB1 in HCM cells subjected to various treatments. C, Effect of PARP1 on the acetylation of HMGB1 was evaluated by Co‐IP assay. D, The viability of HCM cells from different groups was shown. The apoptosis of HCM cells was assessed by flow cytometer (E) and TUNEL (F). G, Protein levels of Bcl‐2, Bax and cleaved caspase‐3 in HCM cells. All results from three independent experiments were expressed as mean ± SD. *P < .05; **P < .01; ***P < .001 vs the indicated group

Since PARP1 was demonstrated to affect LPS‐induced HMGB1 secretion in macrophages via direct interaction with HMGB1,^27^ we further investigated whether PARP1 could affect LPS‐mediated HMGB1 expression in HCM cells. Administration with PJ34, an inhibitor of PARP1, strikingly reduced the mRNA (Figure 2A) and protein (Figure 2B) levels of PARP1 and HMGB1 in LPS‐stimulated HCM cells. Moreover, co‐immunoprecipitation assay indicated that the acetylation of HMGB1 was enhanced after LPS administration. However, PARP1 inhibition attenuated the acetylation of HMGB1 in HCM cell exposure to LPS (Figure 2C). We next sought to investigate the effect of circTLK1 or PARP1 on the viability of HCM cells in response to LPS. Knockdown of circTLK1 significantly inhibited PARP1 and HMGB1 expression in HCM cells (Figure S1). As presented in Figure 2D, the viability of LPS‐exposed HCM cells was significantly enhanced by inhibition of circTLK1 or PARP1. Figure S2A shows the control of the experiment. Additionally, LPS‐induced apoptosis of HCM cells was inhibited by PARP1 or circTLK1 knockdown as detected by flow cytometry (Figure 2E) and TUNEL (Figure 2F). Figure S2B presents the control of apoptosis assay. Furthermore, PARP1 or circTLK1 suppression remarkably reversed LPS‐induced up‐regulation of Bax and cleaved caspase‐3, but down‐regulation of Bcl‐2 in HCM cells (Figure 2G). These findings suggested that circTLK1 or PARP1 silencing inhibited apoptosis of LPS‐stimulated HCM cells.

### 3.3 CircTLK1 or PARP1 suppression represses LPS‐induced oxidative stress damage and mitochondrial dysfunction

**FIGURE 3:**
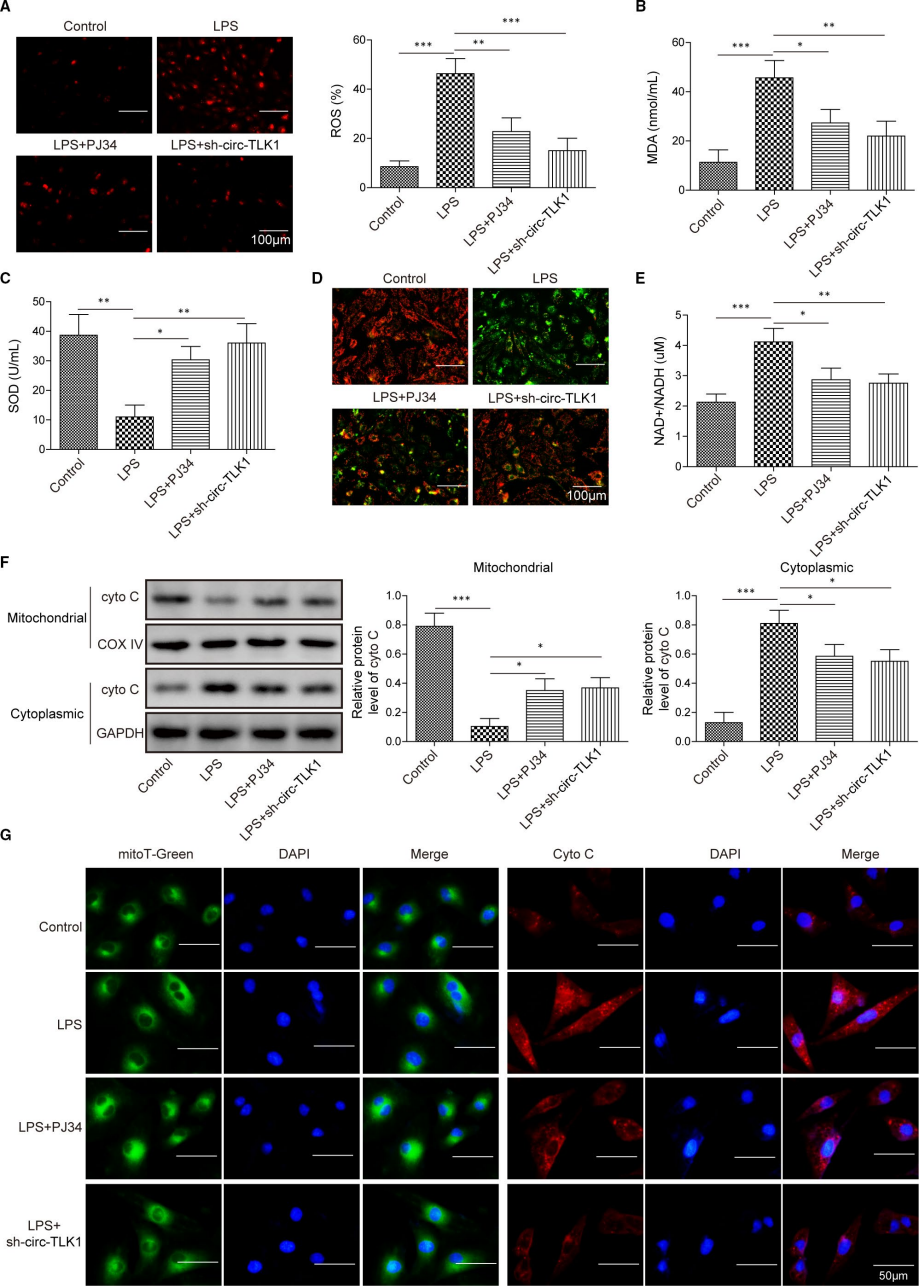
Effect of circTLK1 silencing or PARP1 suppression on LPS‐induced oxidative stress and mitochondrial dysfunction. A, ROS production in HCM cells. The MDA level (B) and SOD activity (C) in HCM cells were determined. D, Mitochondrial membrane potential (∆Ψm) of HCM cells was detected. E, The NAD+/NADH ratio was detected. The distribution of cytochrome C in HCM cells was determined by Western blotting (F) and immunofluorescence staining (G). All results from three independent experiments were expressed as mean  ± SD. *P < .05; **P < .01; ***P < .001 vs the indicated group

Next, we investigated the role of circTLK1 or PARP1 silencing in LPS‐induced oxidative stress in HCM cells. As shown in Figure 3A, the enhanced ROS production in LPS‐stimulated HCM cells was weakened by PARP1 or circTLK1 depletion. In addition, LPS enhanced MDA level (Figure 3B) and reduced SOD activity (Figure 3C) in HCM cells, which were significantly counteracted by PARP1 or circTLK1 inhibition. Figure S2C serves as the control of MDA experiment, and Figure S2D acts as the control of SOD experiment. To further explore the effect of PARP1 or circTLK1 suppression on mitochondrial dysfunction, ∆Ψm was detected by JC‐1 staining. The HCM cells presented decreased ∆Ψm as compared with control cells after challenge with LPS, whereas pre‐treatment with PJ34 or sh‐circTLK1 reversed LPS‐induced loss of ∆Ψm (Figure 3D). Besides, the NAD+/NADH ratio was increased in HCM cells after LPS exposure, which was inhibited by PARP1 or circTLK1 inhibition (Figure 3E). Next, we examined the effect of PARP1 or circTLK1 on distribution of cytochrome C. As assessed by Western blotting (Figure 3F) and immunofluorescence staining (Figure 3G), LPS promoted the release of cytochrome C from mitochondria into cytoplasm; however, inhibition of PARP1 or circTLK1 partly repressed LPS‐induced cytochrome C release. These findings indicated that LPS‐induced oxidative stress damage and mitochondrial dysfunction were attenuated by inhibition of PARP1 or circTLK1.

### 3.4 Inhibition of PARP1 or circTLK1 attenuates mtDNA damage in HCM cells after exposure to LPS

**FIGURE 4:**
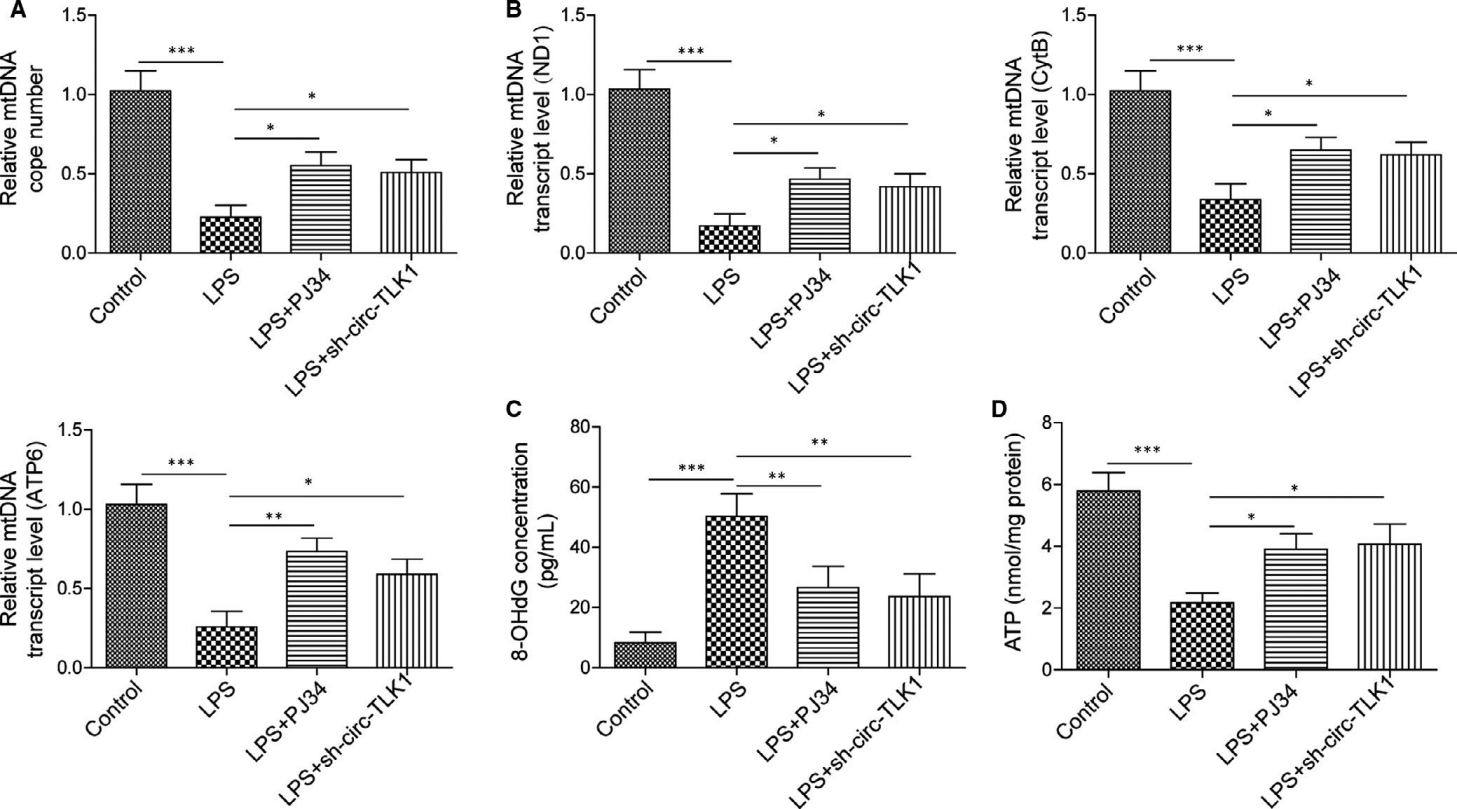
Effect of PARP1 or circTLK1 suppression on mtDNA damage after exposure to LPS. A, The copy number of mtDNA. B, To assess mtDNA transcript levels, mtDNA‐encoded ND1, CytB and ATP6 expression was detected. C, The 8‐OHdG level in HCM cells was determined. D, The ATP level in HCM cells was assessed. All results from three independent experiments were expressed as mean  ± SD. *P < .05; **P < .01; ***P < .001 vs the indicated group

Based on the above results, we further investigated the role of PARP1 or circTLK1 in mtDNA damage. As presented in Figure 4A, the mtDNA copy number was strikingly decreased in LPS‐stimulated HCM cells, as compared with control group, whereas PJ34 or sh‐circTLK1 could abolish this change. Accordantly, inhibition of PARP1 or circTLK1 significantly increased mtDNA transcript levels (ND1, CytB and ATP6) after exposure to LPS (Figure 4B). The control assay is shown in Figure S2F. Moreover, we determined the level of 8‐OHdG, an indicator for DNA oxidative damage. As shown in Figure 4C, LPS stimulation led to an increase in 8‐OHdG level in isolated mitochondria of HCM cells, which was abrogated by PARP1 inhibition or sh‐circTLK1. Figure S2E serves as the control. Importantly, the ATP level was significantly enhanced by PARP1 or circTLK1 inhibition in LPS‐stimulated cells (Figure 4D). These observations showed that inhibition of PARP1 or circTLK1 alleviated LPS‐induced mtDNA damage.

### 3.5 Silencing of circTLK1 restrains oxidative mtDNA damage and apoptosis in LPS‐induced HCM cells via inhibiting PARP1 expression

**FIGURE 5:**
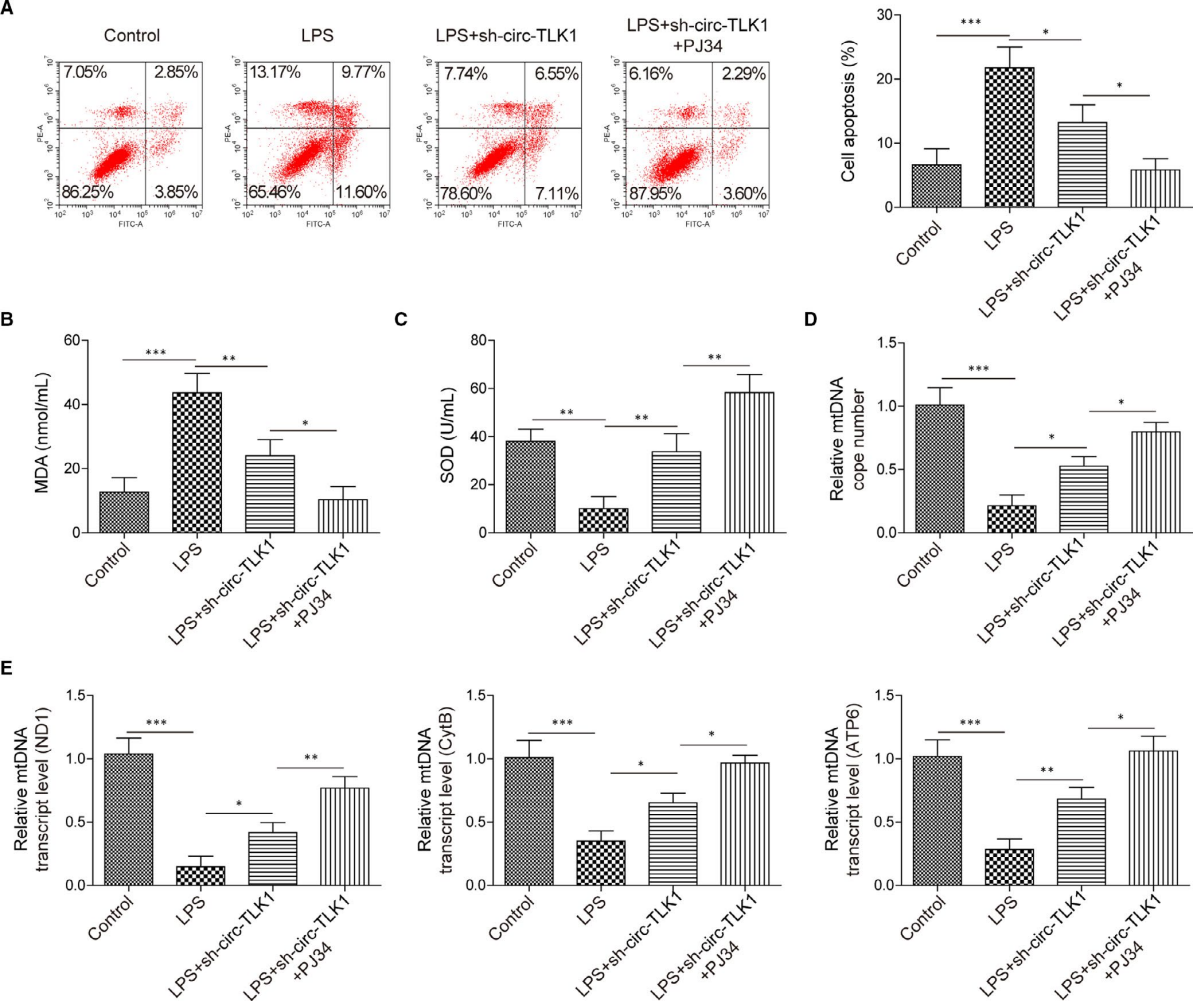
Effect of circTLK1/PARP1 axis inhibition on oxidative mtDNA damage‐mediated apoptosis in LPS‐induced HCM cells. A, The apoptotic rate of HCM cells was detected. The MDA level (B) and SOD activity (C) in HCM cells were evaluated. D, The copy number of mtDNA was detected. E, The mtDNA transcript levels (ND1, CytB and ATP6) were assessed. All results from three independent experiments were expressed as mean  ± SD. *P < .05; **P < .01; ***P < .001 vs the indicated group

To further investigate whether circTLK1 affected LPS‐induced myocardial injury via regulating PARP1, the cells were treated with a combination of PARP1 inhibitor and sh‐circTLK1. As illustrated in Figure 5A, combination with PARP1 inhibitor and sh‐circTLK1 reduced apoptotic rate in HCM cells induced by LPS, as compared with knockdown of circTLK1 alone. In addition, inhibition of circTLK1 decreased MDA level (Figure 5B) and enhanced SOD activity (Figure 5C) in LPS‐exposed HCM cells, which was reinforced when combination with PARP1 inhibition. Furthermore, suppression of circTLK1 increased the mtDNA copy number in LPS‐stimulated HCM cells, and this change was more obvious in sh‐circTLK1+PJ34 group (Figure 5D). We observed the same tendency in mtDNA transcript levels (ND1, CytB and ATP6) (Figure 5E). Therefore, circTLK1 silencing attenuated LPS‐induced mtDNA damage and apoptosis of myocardial cells, which was strengthened by combination with PARP1 inhibitor.

### 3.6 CircTLK1/PARP1 axis participates in oxidative mtDNA damage and cardiomyocyte apoptosis in septic rats

**FIGURE 6:**
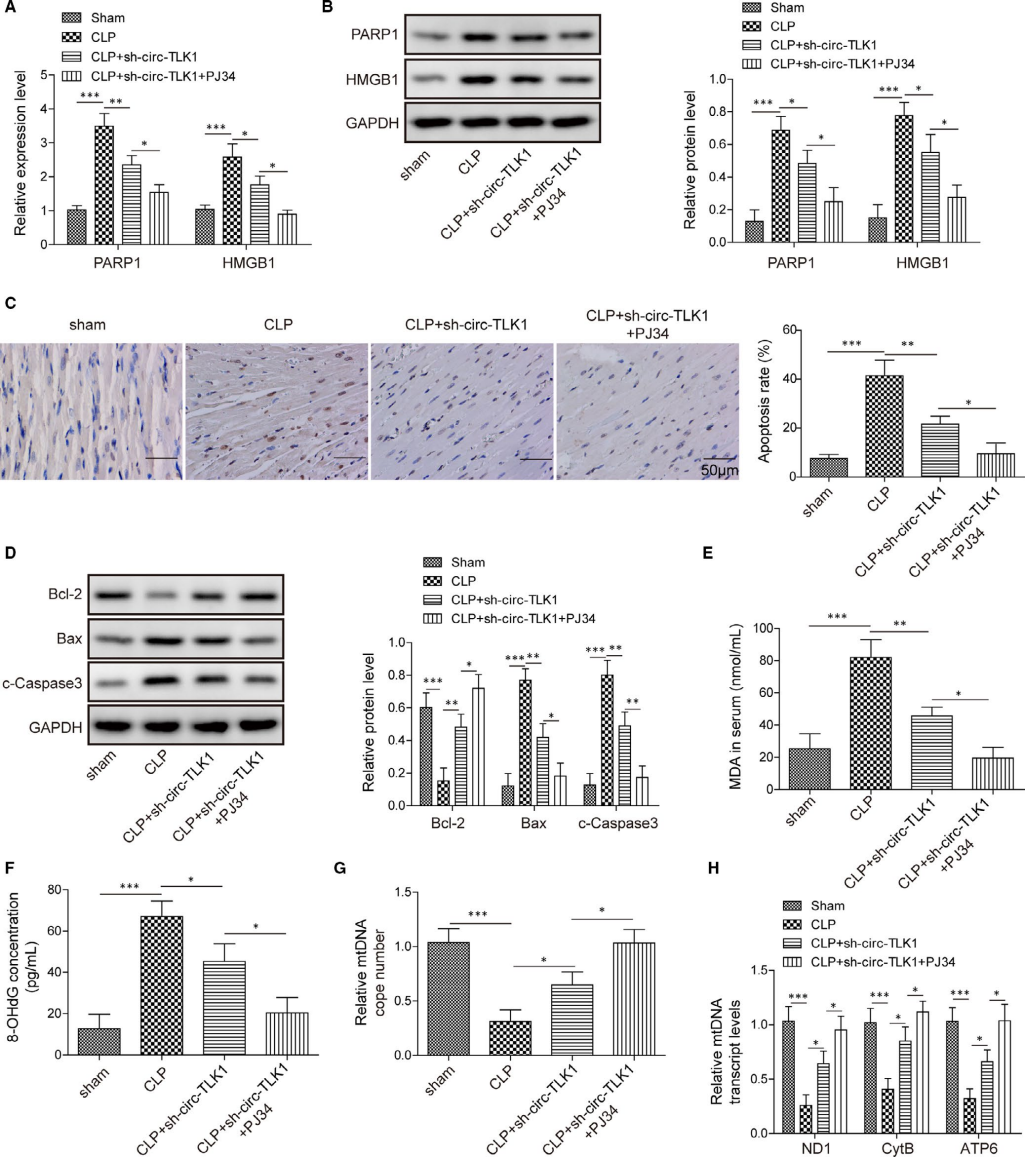
Effect of circTLK1/PARP1 axis on oxidative mtDNA damage–mediated cardiomyocyte apoptosis in septic rats. (A) and (B) PARP1 and HMGB1 expression levels in the myocardial tissues. C, The apoptosis in myocardial tissues. D, The protein levels of Bcl‐2, Bax and cleaved caspase‐3 in myocardial tissues were assessed. The levels of MDA (E) and 8‐OHdG (F) in myocardial tissues were detected. G, The mtDNA copy number in myocardial tissues was determined. H, The mtDNA transcript levels (ND1, CytB and ATP6) in myocardial tissues were determined. All results from three independent experiments were expressed as mean  ± SD. *P < .05; **P < .01; ***P < .001 vs the indicated group

Finally, we carried out in vivo experiments to confirm the regulation of circTLK1/PARP1 axis in oxidative mtDNA damage and cardiomyocyte apoptosis in septic rats. Depletion of circTLK1 significantly reduced the mRNA (Figure 6A) and protein (Figure 6B) levels of PARP1 and HMGB1 in the myocardial tissues of septic rats, which was reinforced by co‐inhibition of PARP1 and circTLK1. As detected by TUNEL, sepsis‐induced apoptosis in myocardial tissues was attenuated by circTLK1 down‐regulation, which was further inhibited in the co‐treatment with PARP1 inhibitor and sh‐circTLK1 (Figure 6C). In addition, circTLK1 silencing enhanced Bcl‐2 expression, while reduced Bax and cleaved caspase‐3 expression in the myocardial tissues of septic rats. These changes were more obvious in combination with PJ34 and sh‐circTLK1 group (Figure 6D). Moreover, as compared with sham group, significant increased MDA and 8‐OHdG levels were observed in septic rats. Co‐suppression of circTLK1 and PARP1 further reduced MDA (Figure 6E) and 8‐OHdG (Figure 6F) levels. Figure S3A is the control of MDA measurement, and Figure S3B is the control of 8‐OHdG examination. Sepsis decreased mtDNA copy number (Figure 6G) and transcript levels (Figure 6H) in the myocardial tissues, which were partly counteracted by circTLK1 knockdown. More importantly, combination with PARP1 inhibitor and sh‐circTLK1 exhibited higher mtDNA copy number and transcript levels than monotherapy. Figure S3C acts as the control of mtDNA copy number detection. Collectively, circTLK1 contributed to oxidative mtDNA damage and cardiomyocyte apoptosis in septic rats via regulating PARP1/HMGB1 axis.

### 3.7 CircTLK1 promotes PARP1 expression via sponging miR‐17‐5p

**FIGURE 7:**
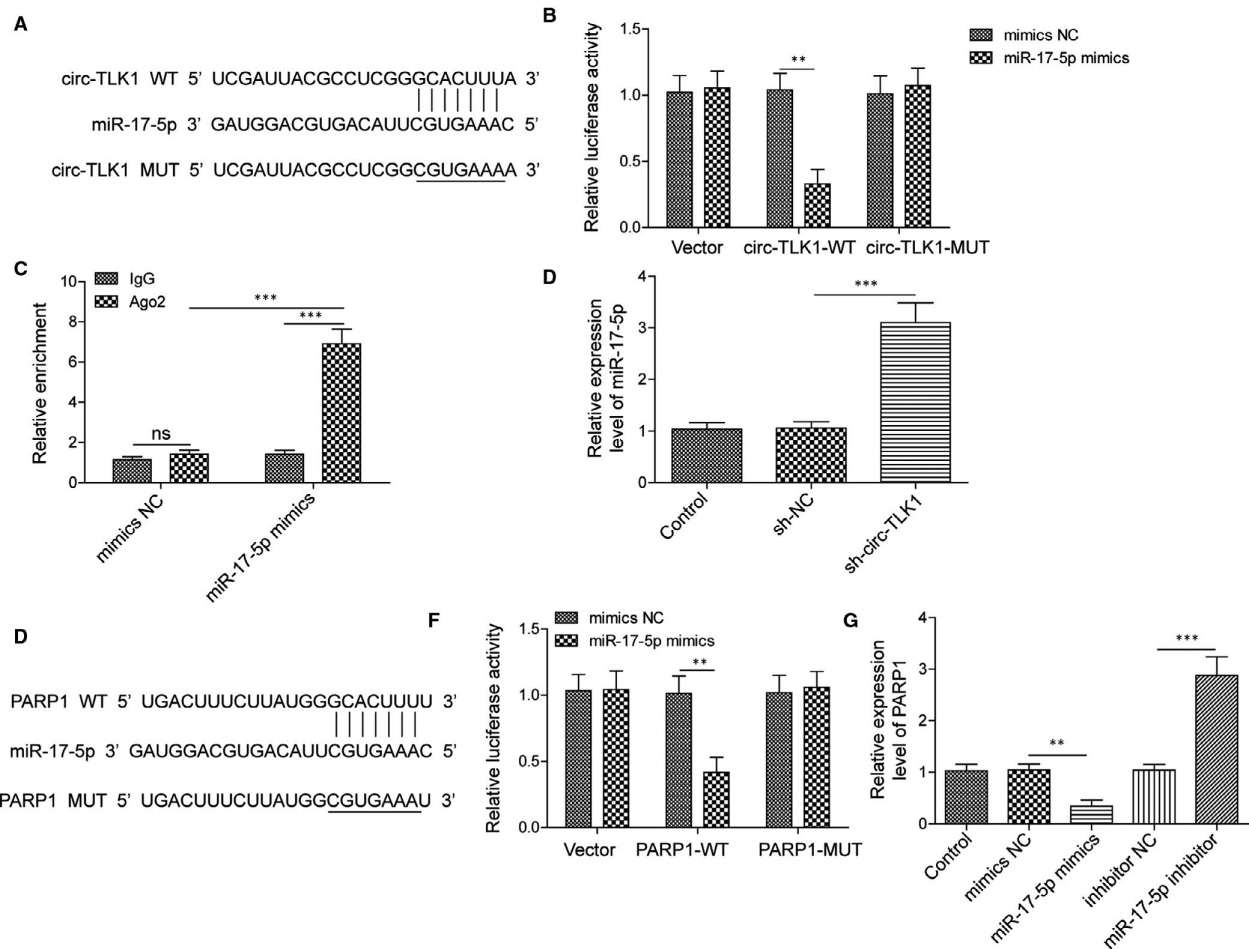
CircTLK1 promotes PARP1 expression via sponging miR‐17‐5p. A, The predicted complementarity between circTLK1 and miR‐17‐5p. B, Dual‐luciferase assay for verifying the relationship between circTLK1 and miR‐17‐5p. C, The direct binding between circTLK1 and miR‐17‐5p was confirmed by RIP assay. D, Expression of miR‐17‐5p in HCM cells was assessed. E, The mRNA expression of PARP1 in HCM cells with various treatments was detected. F, The putative region of complementarity between miR‐17‐5p and PARP1. G, The interaction between miR‐17‐5p and PARP1 was determined by dual‐luciferase assay. All results from three independent experiments were expressed as mean  ± SD. **P < .01; ***P < .001 vs the indicated group

To investigate the mechanism of circTLK1 in regulating PARP1 expression, the complementarity between miR‐17‐5p and circTLK1 was predicted by starBase database (Figure 7A). Further dual‐luciferase reporter system (Figure 7B) and RIP assay (Figure 7C) confirmed the direct interaction between circTLK1 and miR‐17‐5p. Moreover, miR‐17‐5p expression was remarkably increased in circTLK1‐silenced HCM cells (Figure 7D). Additionally, qRT‐PCR analysis showed that the mRNA levels of PARP1 (Figure 7E) and HMGB1 (Figure S4) in HCM cells were increased by miR‐17‐5p inhibitor, but decreased by miR‐17‐5p mimics. By bioinformatics analysis, there is complementarity between miR‐17‐5p and PARP1 (Figure 7F). Further dual‐luciferase reporter system demonstrated that miR‐17‐5p mimics significantly decreased the luciferase activity transfected with PARP1‐WT, but did not affect that transfected with PARP1‐MUT (Figure 7G). These observations indicated that circTLK1 increased PARP1 expression via sponging miR‐17‐5p.

### 3.8 CircTLK1 knockdown attenuates oxidative mtDNA damage and apoptosis via regulating miR‐17‐5p

**FIGURE 8:**
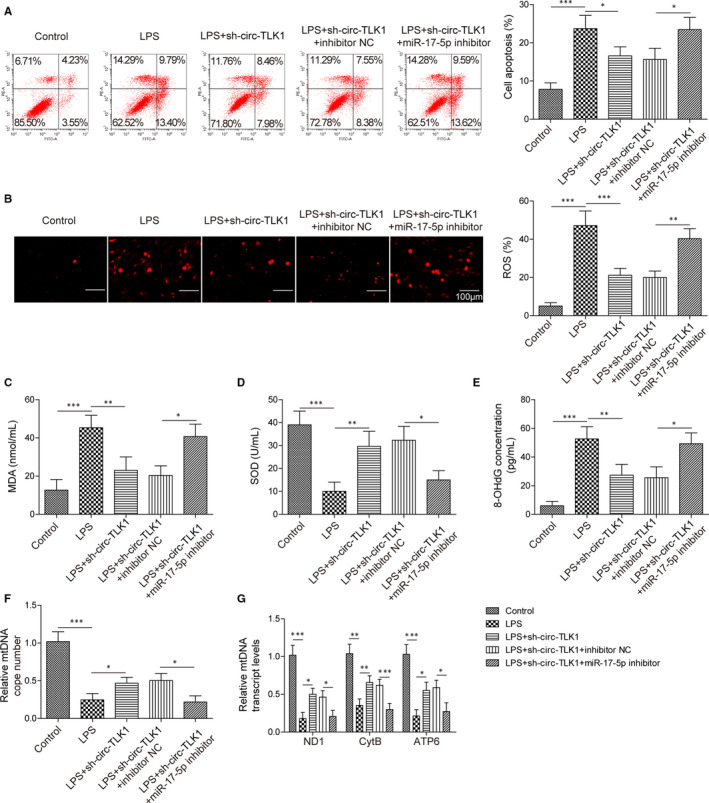
Inhibition of miR‐17‐5p reverses the inhibitory effect of circTLK1 knockdown on oxidative mtDNA damage‐mediated apoptosis. A, The apoptosis of HCM cells from multiple groups was determined. The levels of ROS (B), MDA (C) and SOD (D) in HCM cells were detected. E, The 8‐OHdG level in HCM cells was assessed. F, The mtDNA copy number in HCM cells was evaluated. G, The mtDNA transcript levels (ND1, CytB and ATP6) in HCM cells were detected. All results from three independent experiments were expressed as mean  ± SD. *P < .05; **P < .01; ***P < .001 vs the indicated group

As shown in Figure 8A, miR‐17‐5p inhibitor promoted apoptosis in HCM cells transfected with sh‐circTLK1. As determined by commercial kits, miR‐17‐5p inhibitor enhanced ROS (Figure 8B) and MDA (Figure 8C) levels, while reduced SOD activity (Figure 8D) in circTLK1‐silenced HCM cells. In addition, inhibition of miR‐17‐5p enhanced 8‐OHdG level after the transfection with sh‐circTLK1 (Figure 8E). miR‐17‐5p inhibitor reduced the mtDNA copy number (Figure 8F) and transcript levels (Figure 8G) in circTLK1‐depleted HCM cells. Thus, circTLK1 affected oxidative mtDNA damage and apoptosis via regulating miR‐17‐5p.

## 4 DISCUSSION

**FIGURE 9:**
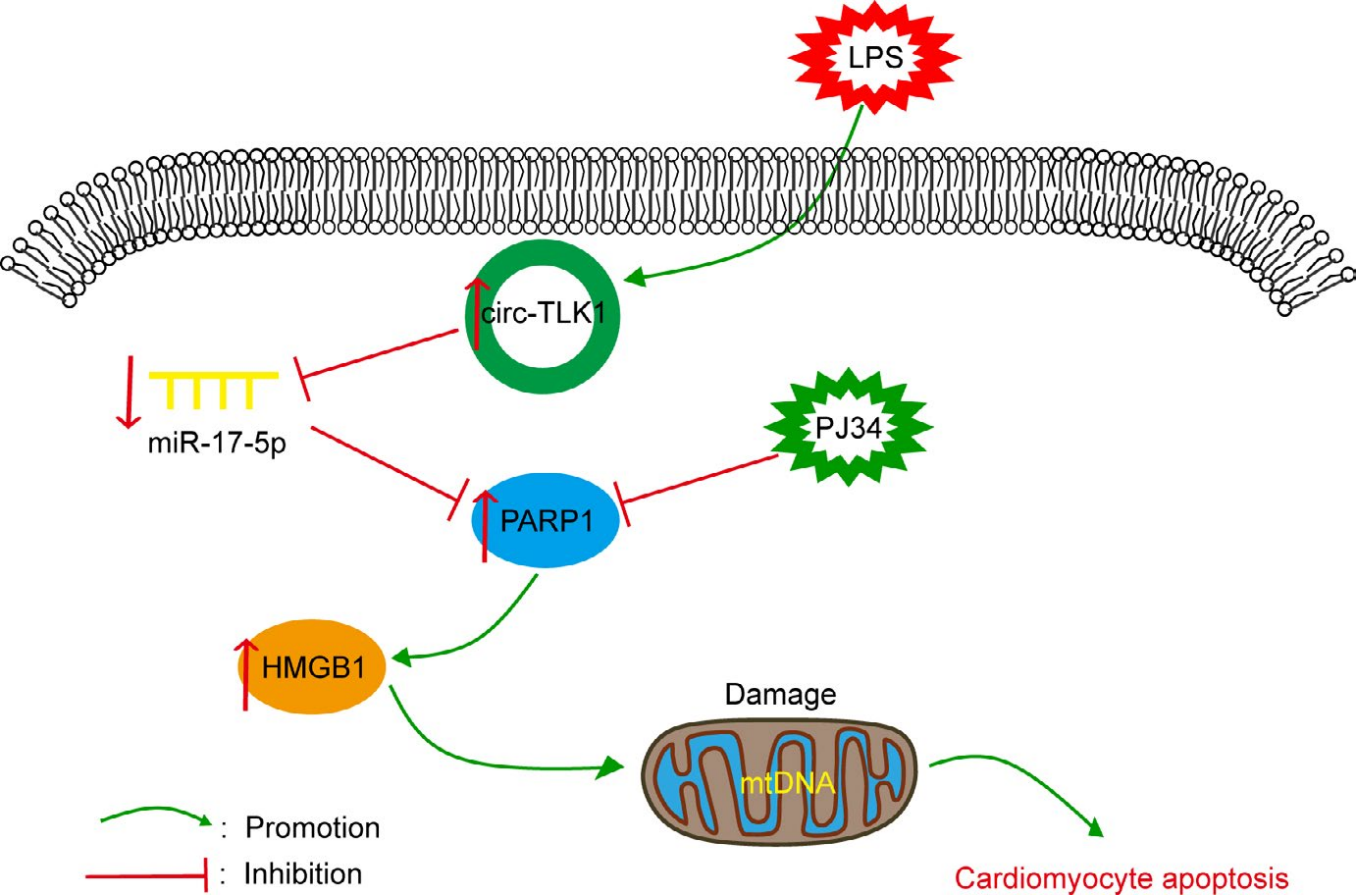
A schematic overview of the role of circTLK1/miR‐17‐5p/PARP1/HMGB1 axis in LPS‐induced mtDNA oxidative damage and subsequent cardiomyocyte apoptosis

This study for the first time revealed that silencing of circTLK1 could inhibit PARP1 and HMGB1 expression in septic cardiomyopathy. Moreover, the in vitro and in vivo data showed that inhibition of circTLK1 suppressed oxidative mtDNA damage, mitochondrial dysfunction and subsequent apoptosis during septic cardiomyopathy via inhibiting PARP1/HMGB1 axis by modulating miR‐17‐5p. Our findings identify circTLK1/miR‐17‐5p/PARP1/HMGB1 axis as a potential therapeutic target for septic cardiomyopathy. The schematic of this study is summarized in Figure 9.

Dysregulation of gene expressions takes part in the development of septic cardiomyopathy. The latest evidence has showed that circRNAs are regarded as key regulators of sepsis.^36^ To comprehensively understand the pathogenesis of septic cardiomyopathy, we focused on circTLK1. Our results showed that circTLK1 was up‐regulated in LPS‐exposed cardiomyocytes, indicating that circTLK1 might be involved in septic cardiomyopathy progression. PARP1 has been shown to participate in sepsis development, and knockdown of PARP1 could protect against sepsis‐induced acute lung injury^37^ and cardiac dysfunction.^38^ Several observations have indicated that enhance expression of HMGB1 contributed to cardiac dysfunction during sepsis.^39^, ^40^ Interestingly, PARP1 has been demonstrated to promote the secretion of HMGB1 in sepsis.^27^ Consistent with these studies, we observed a significant increase in PARP1 and HMGB1 expression in the in vitro and in vivo models of sepsis, suggesting that PARP1/HMGB1 axis might play pivotal roles in septic cardiomyopathy.

It has been recognized that the production of ATP by mitochondria maintains the metabolism and function of myocardial cells. Mitochondrial dysfunction leads to energy depletion and consequent myocardial injury during sepsis.^41^ Oxidative stress has been considered as another player in the progression of septic cardiomyopathy. Previous studies showed that the excessive generation of ROS may cause oxidative stress damage to the mitochondria of cardiomyocytes during septic cardiomyopathy.^12^, ^42^ Multiple antioxidant enzymes (eg SOD) are crucial to protect against mitochondrial oxidative stress. Mounting evidence has proved that sepsis‐induced myocardial injury could be attenuated by inhibiting oxidative stress and mitochondrial dysfunction.^43^, ^44^ A recent research showed that inhibition of PARP1 delayed the progression of Chagasic cardiomyopathy through improving mitochondria function and maintaining oxidant/antioxidant balance.^45^ Dong et al suggested that the release of HMGB1 caused mitochondrial oxidative damage in ventilator‐induced lung injury.^46^ Besides, silencing of circTLK1 protected against cardiomyocyte apoptosis during myocardial ischaemia/reperfusion injury.^17^ Consistent with these observations, we demonstrated that circTLK1 knockdown decreased PARP1 and HMGB1 expression in cardiomyocytes. Moreover, circTLK1 or PARP1 depletion reduced the production of ROS and MDA, increased SOD activity and improved mitochondrial dysfunction as evidenced by enhancing ∆Ψm, lowering NAD+/NADH ratio and inhibiting cytochrome C release into the cytosol during septic cardiomyopathy. Co‐inhibition of circTLK1 and PARP1 enforced these beneficial effects. Thus, circTLK1 inhibition improved sepsis‐induced oxidative stress damage and mitochondrial dysfunction by inhibiting PARP1/HMGB1 axis.

mtDNA integrity is essential for the maintenance of mitochondrial function. MtDNA is especially vulnerable because of its location nearing the place where excessive ROS are produced, as well as the lack of protective histones.^8^ More importantly, the repair of damaged mtDNA is less efficient than nuclear DNA due to the missing of nucleotide excision repair.^47^ The implication of mtDNA damage in the progression of septic cardiomyopathy has been well documented.^48^ Vitamin E could protect against sepsis‐induced cardiac injury via suppression of oxidative mtDNA damage.^12^ In the present study, sepsis led to decrease in mtDNA copy number and transcript levels. Silencing of circTLK1/PARP1 axis could effectively relieve myocardial mtDNA damage during sepsis. Subsequently, the intrinsic apoptosis pathways can be activated by mtDNA damage–mediated mitochondrial dysfunction.^49^ Loss of ∆Ψm may result in cytochrome C release into the cytoplasm and then induce caspase‐3 activation–mediated apoptosis.^50^ The balance between pro‐apoptosis protein Bax and anti‐apoptosis protein Bcl‐2 determines apoptosis.^51^ According to our results, sepsis‐induced myocardial apoptosis was significantly repressed by inhibition of circTLK1 or PARP1. Collectively, circTLK1 led to mtDNA oxidative damage and subsequent cardiomyocyte apoptosis during septic cardiomyopathy through activating PARP1/HMGB1 axis.

Sponging miRNAs have been recognized as one of functional mechanisms of circRNAs.^18^ For example, circTLK1 facilitated renal cell carcinoma cell proliferation, migration and invasion via sponging miR‐136‐5p.^52^ Silencing of circTLK1 attenuated myocardial ischaemia/reperfusion injury by sponging miR‐214.^17^ Up‐regulation of miR‐17‐5p suppressed inflammation in sepsis, which was suggested as a therapeutic target for sepsis.^53^ In the current study, the complementarity between circTLK1 and miR‐17‐5p was predicted by bioinformatics analysis. Further dual‐luciferase reporter assay and RIP assay verified the direct binding between circTLK1 and miR‐17‐5p. circTLK1‐mediated negative regulation of miR‐17‐5p expression was confirmed in HCM cells. Interestingly, we also observed the region of complementarity between miR‐17‐5p and PARP1, which was further validated by dual‐luciferase reporter assay. Inhibition of miR‐17‐5p abolished the beneficial effect of sh‐circTLK1 on oxidative mtDNA damage. Our findings revealed that circTLK1 sponged miR‐17‐5p to induce oxidative mtDNA damage in septic cardiomyopathy via targeting PARP1.

We are aware of the fact that this study has some limitations. First, the expression of PARP1 and HMGB1 was inhibited only using chemical inhibitors. However, the effect of genetical inhibition of PARP1 and HMGB1 remains unclear. Second, the downstream mechanisms of PARP1/HMGB1 axis in septic cardiomyopathy have not been explained. In our future research, these issues will be specially focused on.

Taken together, our data indicated that circTLK1 sponged miR‐17‐5p to promote PARP1/HMGB1 axis–mediated oxidative mtDNA damage, mitochondrial dysfunction and consequent myocardial apoptosis, which uncover the pathological mechanisms of septic cardiomyopathy. Our findings provide evidence that targeting circTLK1/miR‐17‐5p/PARP1/HMGB1 axis confers an effective protection against sepsis‐induced myocardial injury.

## CONFLICT OF INTEREST

The authors confirm that there are no conflicts of interest.

## AUTHOR CONTRIBUTIONS

**Yu Qiu:** Conceptualization (equal); Funding acquisition (equal); Methodology (equal); Writing‐original draft (equal). **Ying Yu:** Data curation (equal); Formal analysis (equal); Validation (equal). **Xiao‐Mei Qin:** Data curation (equal); Formal analysis (equal); Investigation (equal). **Tao Jiang:** Formal analysis (equal); Investigation (equal); Validation (equal); Visualization (equal). **Yan‐Fang Tan:** Investigation (equal); Methodology (equal); Resources (equal); Writing‐original draft (equal). **Wen‐Xian Ouyang:** Formal analysis (equal); Software (equal); Visualization (equal); Writing‐original draft (equal). **Zheng‐Hui Xiao:** Conceptualization (equal); Methodology (equal); Project administration (equal); Resources (equal); Supervision (equal); Writing‐review & editing (equal). **Shuang‐Jie Li:** Conceptualization (equal); Methodology (equal); Project administration (equal); Supervision (equal); Writing‐review & editing (equal).

## ETHICS APPROVAL

All experiments performed were approved by the Animal Care and Use Committee of Hunan Children’s Hospital.

## Supporting information

Fig S1

Fig S2

Fig S3

Fig S4

## Data Availability

All data generated or analysed during this study are included in this published article.
