# Supplementary figures and images for: CircTLK1 modulates sepsis‐induced cardiomyocyte apoptosis via enhancing PARP1/HMGB1 axis–mediated mitochondrial DNA damage by sponging miR‐17‐5p
Source: J Cell Mol Med. 2021 Aug 19;25(17):8244–60. doi: 10.1111/jcmm.16738 (PMC8419196; doi:10.1111/jcmm.16738)

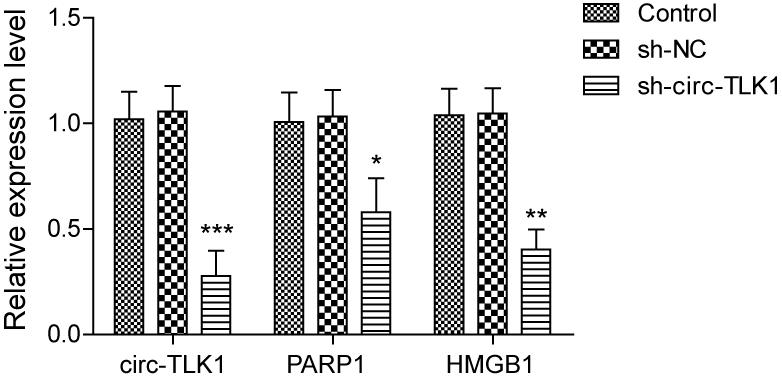

Supplement: Supplementary file 1 — Fig S1 [file JCMM-25-8244-s001.tif]

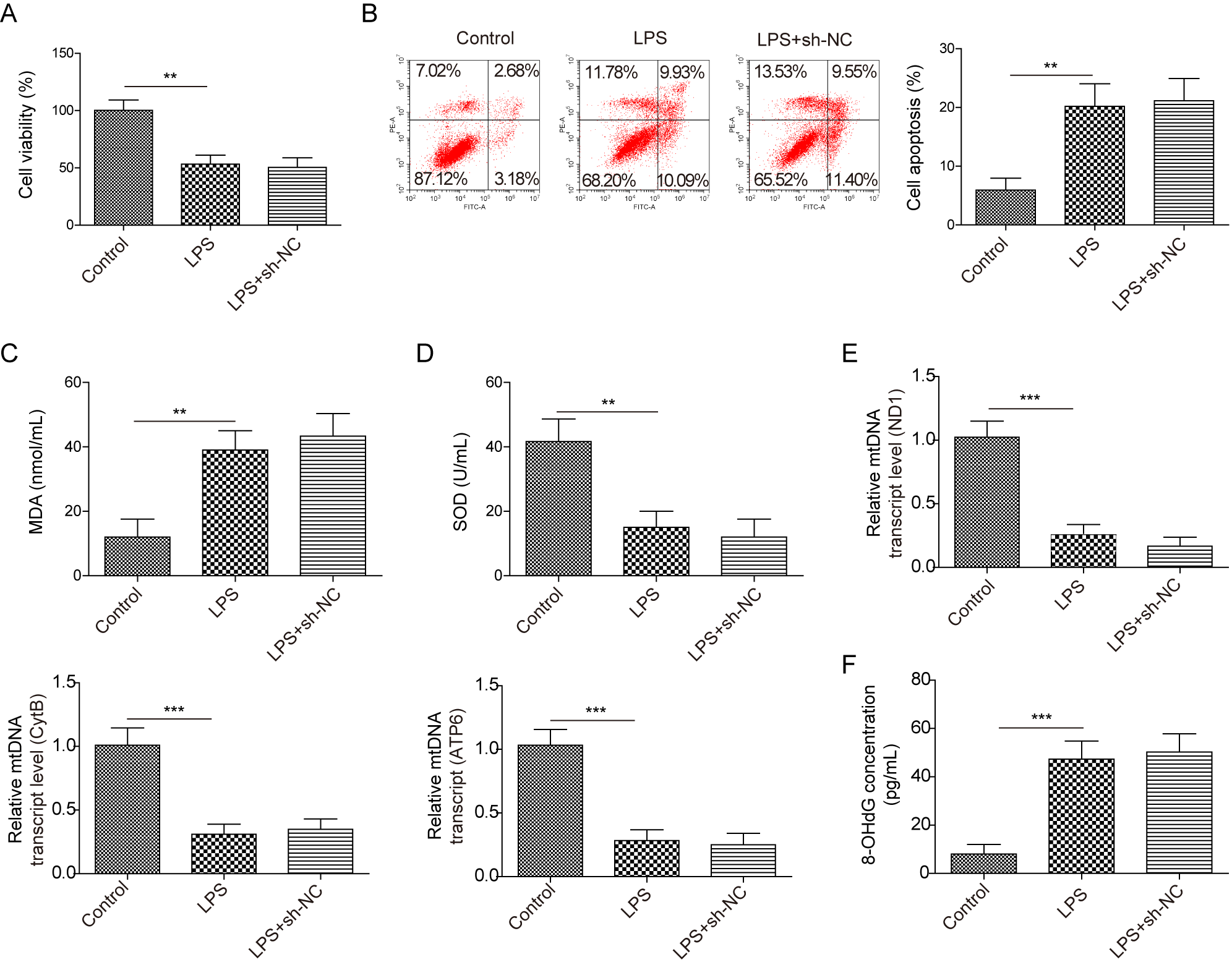

Supplement: Supplementary file 2 — Fig S2 [file JCMM-25-8244-s002.tif]

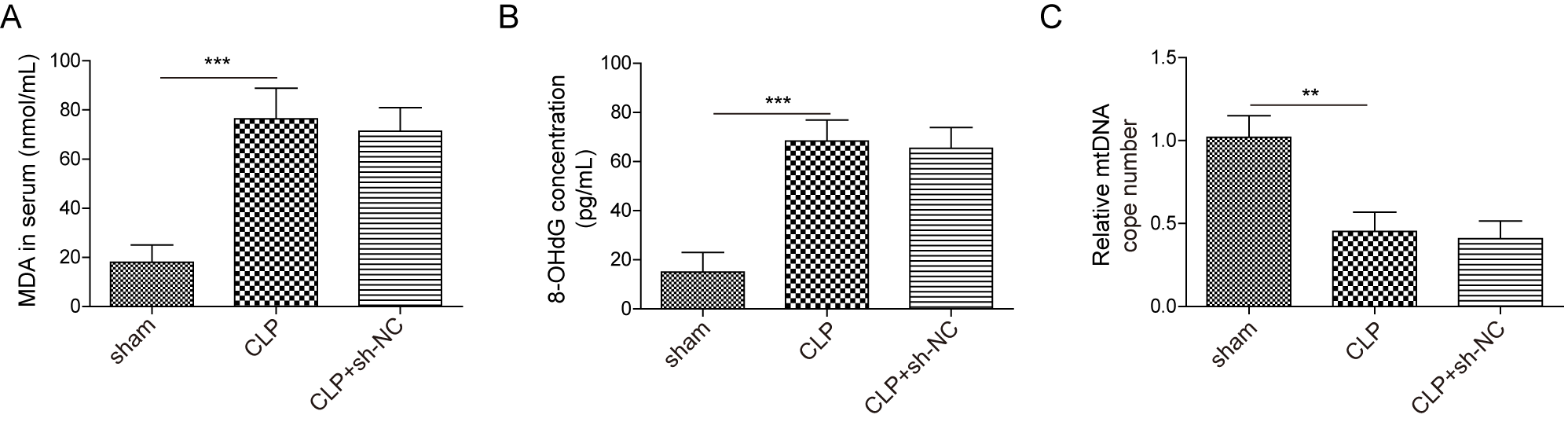

Supplement: Supplementary file 3 — Fig S3 [file JCMM-25-8244-s003.tif]

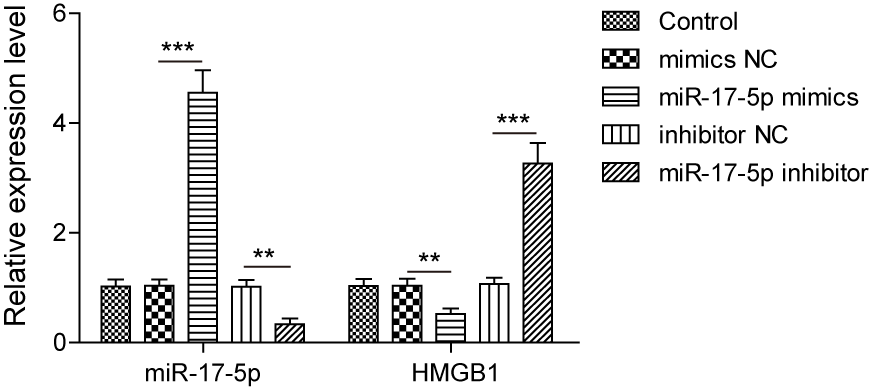

Supplement: Supplementary file 4 — Fig S4 [file JCMM-25-8244-s004.tif]
